# Clinical Significance of Nectins in HCC and Other Solid Malignant Tumors: Implications for Prognosis and New Treatment Opportunities—A Systematic Review

**DOI:** 10.3390/cancers15153983

**Published:** 2023-08-05

**Authors:** Jakub Klekowski, Dorota Zielińska, Adriana Hofman, Natalia Zajdel, Paweł Gajdzis, Mariusz Chabowski

**Affiliations:** 1 Department of Nursing and Obstetrics, Division of Anesthesiological and Surgical Nursing, Faculty of Health Science, Wroclaw Medical University, 50-367 Wroclaw, Poland; klekowski.jakub@gmail.com; 2 Department of Surgery, 4th Military Teaching Hospital, 50-981 Wroclaw, Poland; doorotaz@gmail.com; 3 Student Research Club No 180, Faculty of Medicine, Wroclaw Medical University, 50-367 Wroclaw, Poland; ada.hofman@op.pl (A.H.); nataliazajdel13@gmail.com (N.Z.); 4 Department of Clinical and Experimental Pathology, Faculty of Medicine, Wroclaw Medical University, 50-367 Wroclaw, Poland; pgajdzis@protonmail.com; 5 Department of Pathomorphology, 4th Military Teaching Hospital, 50-981 Wroclaw, Poland

**Keywords:** overall survival, prognostic factors, nectins, cancers

## Abstract

**Simple Summary:**

Nectins are attracting increased interest as potential markers for prognosis. In this review, we summarize the results of studies concerning the prognostic utility of the nectin family in different cancers. The reports on nectins-1-3 are ambiguous—both the upregulation and loss of expression could be a prognostic indicator. Nectin-4 is quite consistently overexpressed in tumors, which generally correlates with a worse prognosis. The data on nectins in hepatocellular carcinoma (HCC) are rather scarce and require further research. New treatments based on nectin-4 are currently being introduced, opening up possibilities for new lines of therapy.

**Abstract:**

The nectin family comprises four proteins, nectin-1 to -4, which act as cell adhesion molecules. Nectins have various regulatory functions in the immune system and can be upregulated or decreased in different tumors. The literature research was conducted manually by the authors using the PubMed database by searching articles published before 2023 with the combination of several nectin-related keywords. A total of 43 studies were included in the main section of the review. Nectins-1–3 have different expressions in tumors. Both the loss of expression and overexpression could be negative prognostic factors. Nectin-4 is the best characterized and the most consistently overexpressed in various tumors, which generally correlates with a worse prognosis. New treatments based on targeting nectin-4 are currently being developed. Enfortumab vedotin is a potent antibody–drug conjugate approved for use in therapy against urothelial carcinoma. Few reports focus on hepatocellular carcinoma, which leaves room for further studies comparing the utility of nectins with commonly used markers.

## 1. Introduction

Cancers, along with cardiovascular diseases (CVD), are the leading causes of death worldwide. According to an analysis of recent trends, cancers may surpass CVD as the leading cause of death in most countries within this century [1]. The management and care of cancer patients is a great economic burden for healthcare systems and the expenditure rise is expected to continue [2]. Although the novel treatment methods—including surgery, chemotherapy and radiation—and the accuracy of diagnostic measures of many cancers have been advancing rapidly in the past few decades, the prognosis, as well as the patients’ quality of life, still remain unsatisfactory for many cancers [3,4,5,6,7]. Therefore, there is undoubtedly a constant need for improvement in the approach to cancer management.

Nectins and necl (nectin-like molecules) are a broad family of type I single-pass transmembrane proteins belonging to the Ca^2+^-independent immunoglobulin superfamily. The nectin family comprises four proteins, nectin-1 to -4, which act as cell adhesion molecules (CAMs). All nectins can also be searched using alternate names—poliovirus receptor-related protein 1–4 (PVRL1–4)—however, nectins-1 to -3 are identified in the cluster of differentiation (CD) using numbers 111–113 [8]. What differentiates nectins from necl is that nectins have the ability to bind afadin (an actin filament-binding protein) through its C-terminal sequence on their cytoplasmic tail. The necl family is composed of five members with consecutive numbers from 1 to 5 [9,10].

Nectins’ expression differs depending on the tissue; however, nectins-1–3 are generally expressed in adult tissues, while nectin-4, in normal conditions, is found mostly in placenta and embryonic tissues. The main functionality of nectins is their ability to form homophilic as well as heterophilic associations. These cell-to-cell adhesions mediated by nectins are substantial in the development on many stages and in maintaining tissue homeostasis. Homophilic interactions are generally restricted to performing adhesive functions. Furthermore, in heterophilic associations, nectins can bind each other: nectin-4 to nectin-1, nectin-1 to nectin-3 and nectin-3 to nectin-2. Nectins also bind selectively with necl family members. Binding afadin further recruits a- and b-catenins and cadherins to the binding site, which results in the formation of adherens junctions. Apart from homo- and heterophilic interactions within the nectin family, there are other functions that nectins exhibit by binding specific proteins—especially immune modulation, cell survival, growth and proliferation signaling, and viral entry. Among others, the physiological functions of nectins include angiogenesis, leukocyte vascular migration and other cells’ migration [9,11,12,13]

Members of the nectins and necl families have a regulatory function in T-lymphocytes and natural killer (NK) cells. Studies in recent years have shed light on nectins’ and necl’s interactions with T-cell immunoglobulin and ITIM domain (TIGIT) family receptors, which determine the activity of immune cells. The TIGIT family receptors comprise TIGIT, CD226 (or DNAM-1, DNAX-associated molecule 1), CD96 and CD112R. Nectins-2,-3 and -4 have the ability to bind TIGIT; additionally, nectin-2 can interact with CD226, while nectin-1 interacts with CD96. Notably, necl-5 recognizes TIGIT, CD226 and CD96; therefore, nectin-2/necl-5 are engaged in specific stimulatory/inhibitory activity with CD226/TIGIT. The interactions between these receptors and their ligands determine the immunological response; thus, the expression of these molecules will be responsible for the profile of immunological activity. The disturbance of balance within this network leads to an increased susceptibility to infection and neoplasm development. Interactions within this network are somewhat similar to those involving CD28 and T-lymphocyte-associated protein 4 (CTLA-4), which is one of the immune-checkpoint receptors along with the programmed cell-death protein 1 (PD-1). TIGIT, CD226 and CD96 are now broadly explored for possible targets of immunotherapy for cancer [9,13,14,15,16].

Nectins were found to have different expressions in various cancers. Most nectins are observed to be overexpressed in malignancies like colorectal cancer (CRC), breast cancer or prostate cancer. Nectin-4 stands out among the other family members since it is widely expressed in fetal tissues, placenta and multiple cancers, while it is rarely found in developed tissues. This characteristic was the basis for further studies on this nectin’s utility in determining the diagnosis, prognosis and possible treatment targets for cancer. Currently, Enfortumab vedotin—a monoclonal antibody–drug conjugate targeting nectin-4—is used as another tool for treatment of urothelial carcinoma [12,17,18]. The characteristics of the nectin family have been summarized below (Table 1).

The aim of this review was to evaluate the current knowledge on nectins’ significance in terms of the prognosis of different cancers and also their utility for diagnostic purposes. Additionally, we took a closer look at emerging cancer treatment methods based on nectins. The majority of the reviews focus on drug therapies based on targeting nectins or concern only selected cancers and nectins; therefore, this review stands out in the current literature.

## 2. Literature Research

**Figure 1:**
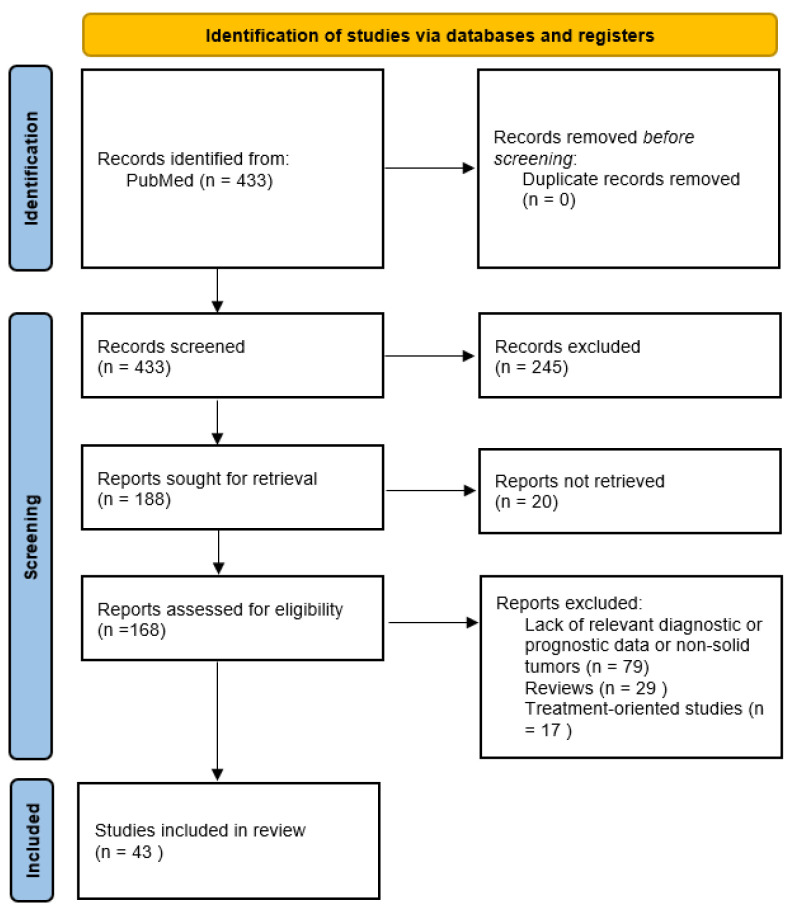
PRISMA chart of literature research.

The literature research was conducted manually, with the authors searching the PubMed database for eligible articles published before 2023 with the following keywords: ‘nectin cancer’, ‘nectin-1 cancer’, ‘nectin-2 cancer’, ‘nectin-3 cancer’, ‘nectin-4 cancer’, ‘CD111 cancer’, ‘CD112 cancer’, ‘CD113 cancer’, ‘PVRL-4 cancer’. The records were screened for further assessment and were excluded if the article did not target the topic of nectin utilization in the prognosis, diagnosis and treatment of cancers. After retrieval, all the articles were manually assessed to determine their eligibility to be included in the review. The exclusion criteria were as follows: written in a language other than English; took the form of a case report or review; irrelevant to the topic or contained insufficient data. In the end, 43 studies were included in the main section of the review concerning the prognostic and diagnostic utility of nectins. Specific numbers are presented in the PRISMA chart in Figure 1.

## 3. Nectin-1

Nectin-1 has various patterns of expression in different cancers. The following reports present nectin-1 as a potential biomarker in several malignancies (Table 2).

In a murine-based study on hepatocellular carcinoma (HCC), Chiu et al. reported a high expression of nectin-1 in cancer cells and reduced tumor growth in mice after nectin-1 knock-down, which was linked to the decreased recruitment of TIGIT in these tumors [19]. Wang et al. explored nectins’ involvement in HCC on human tissues. The authors studied 28 patients’ paired cancer-adjacent non-cancerous tissue samples and expanded the study with gene-expression data and DNA-methylation data from The Cancer Genome Atlas (TCGA) liver hepatocellular carcinoma datasets. The reported expression of nectin-1 was high in the tumor tissue compared to the adjacent non-cancerous tissue. Furthermore, the authors found that up-regulation of nectin-1 correlated with a poor prognosis for patients and was closely related to the higher stages and grades of HCC. In addition, the researchers concluded that nectin-1 can promote the migration and proliferation of HCC cells. They considered the potential of nectin-1 as a marker and a target for treatment [20].

**Table 2:** Nectin-1 expression and impact on prognosis in cancers.

Authors	Cancer	Material	Results and Conclusions
Wang et al. [20]	HCC	28 paired samples—cancerous and non-cancerous tissue. Additionally, gene expression data (TCGA)	High nectin-1 expression in cancer compared to adjacent tissues. Up-regulation of nectin-1 correlated with a worse prognosis and more advanced disease.
Martin et al. [21]	BrC	133 breast cancer tissues, 27 control cancer-free breast tissues	Nectin-1 expression was elevated in tumors, especially tumors with a higher TNM stage, tumor grade and NPI, ductal carcinomas and ER+ tumors. Higher expression was an indicator of poor survival (but the difference was not significant).
Yamada et al. [22]	PDAC	Carcinoma-associated-fibroblasts (CAF) in 258 samples	Nectin-1 expressed in 64 tumors, correlated with lymph node metastasis (N1-2), IIB-IV TNM stage, perineural invasion, tumor location in the pancreatic head and shorter OS.
Izumi et al. [23]	PAC	49 cancer samples	Nectin-1 was highly expressed in the cancer tissues, but no correlation with OS was found.
Tampakis et al. [24]	CRC	111 tissue samples	High expression of nectin-1 in 59.5% of tumors. Significantly higher expression in advanced tumors (stage III–IV). Lymph node metastasis correlates with a high expression of nectin-1. Nectin-1 was an independent prognostic factor of disease recurrence and death.
Takahashi et al. [25]	GC	406 patients’ data from TCGA Samples resected surgically and endoscopically (89 and 81, respectively)	Low expression of nectin-1 indicates poor survival (median 25.6 months vs. 55.4 months in the case of a high expression).
Ahn et al. [26]	BUC	165 tissue samples	Nectin-1 expression was an independent predictive factor of worse OS.
Han et al. [27]	LGG	TCGA, CGGA and REMBRAND databases	Low expression of nectin-1 was a predictor of worse prognosis.
Kälin et al. [28]	mCRPC	57 serum samples	A higher level of nectin-1 was one of predictors of worse survival (in the set of 5 proteins).

HCC—hepatocellular carcinoma; BrC—breast cancer; PDAC—pancreatic ductal adenocarcinoma; PAC—pancreatic adenocarcinoma; CRC—colorectal cancer; GC—gastric cancer; BUC—bladder urothelial carcinoma; LGG—low-grade glioma; mCRPC—metastatic castration resistant prostate cancer.

Martin et al. studied nectins’ expression in breast cancer samples and showed increased expression of all nectins in the tumors, although the difference was not significant. In terms of nectin-1, the researchers found an even greater elevated expression in node-positive tumors. Moreover, alongside the increasing TNM stage, tumor grade and Nottingham Prognostic Indicator (NPI), the expression of nectin-1 also increased. Estrogen receptor-positive (ER+) tumors expressed nectin-1 more strongly than ER-negative tumors. Apart from that, the expression in ductal carcinomas was higher than in other types of tumors. The elevated expression of nectin-1 implied poorer survival and nectin-1 was the only nectin which was increased in metastatic disease [21].

In pancreatic ductal adenocarcinoma (PDAC), Yamada et al. evaluated nectin-1 expression in carcinoma-associated fibroblasts (CAF). A total of 258 tissue samples were studied by means of immunohistochemistry (IHC). In 64 patients, nectin-1 expression in CAF was detected and it was correlated with lymph node metastasis (N1-2), IIB-IV TNM stage, perineural invasion, tumor location in the pancreatic head and, most importantly, with shorter overall survival (OS). The median OS for patients with nectin-1 expression in CAF was significantly lower compared to patients without nectin-1 expression in CAF (19 months vs. 29 months, *p* = 0.003). Multivariate analysis showed that nectin-1 expression in CAF was an independent prognostic factor of worse OS [22]. Conversely, in the study by Izumi et al., nectin-1, although generally expressed in the pancreatic adenocarcinoma studied (based on IHC expression scores), had no impact on OS in the group in question [23].

Nectin-1 expression in colorectal cancer (CRC) was assessed via IHC by Tampakis et al. The authors examined 111 tissue samples of resected CRC. Strong expression of nectin-1 was detected in the majority of samples (59.5%). Reportedly, nectin-1 was not expressed in less than 10% of the samples. In samples from highly advanced CRC (stage III–IV), nectin-1 expression was significantly higher than in lower-stage (I–II) tumors (*p* = 0.012). Lymph node metastasis was proved to be associated with higher nectin-1 expression (*p* = 0.007). Other tumor features, like T stage, metastasis, the location of the tumor, the grade of cellular differentiation, vascular and peri-neural invasion and mucinous histologic subtype, were not linked to nectin-1 expression. Within 36 months after surgery, 55.7% of patients with a high nectin-1 expression presented disease recurrence or death versus 82.1% of patients with a low nectin-1 expression (*p* = 0.014). The authors identified nectin-1 as an independent prognostic factor of disease recurrence and death in CRC [24].

In another gastrointestinal cancer, Takahashi et al. explored nectin-1 expression using IHC in gastric cancer (GC). The authors compared TCGA data on gastric adenocarcinoma (406 patients) with surgically and endoscopically (89 and 81 samples, respectively) resected tissues of GC. According to the data from TCGA, patients with tumors with low nectin-1 expression had poorer survival rates than patients with highly expressing tumors. Another reported finding was that nectin-1 expression was significantly decreased in diffuse-type GC compared with intestinal-type GC [25].

Nectin-1 prognostic significance was analyzed in bladder urothelial carcinoma (BUC) by Ahn et al. Their research involved 165 cancer samples on which IHC staining was performed. The expression of nectin-1 was associated with worse OS and shorter relapse-free survival. Multivariate analysis proved nectin-1 to be an independent prognostic factor of worse OS [26].

Han et al. explored the role of nectins in the prognosis of low-grade glioma (LGG). The authors analyzed several databases: TCGA, Chinese Glioma Genome Atlas (CGGA) and Repository of Molecular Brain Neoplasia Data (REMBRAND). Low expression of nectin-1 was an indicator of worse prognosis in all three databases [27].

In metastatic castration-resistant prostate cancer (mCRPC), Kälin et al. evaluated multiple proteins in patient serum samples in order to create a set of new prognostic markers. The authors extracted a survival predictor composed of five proteins which contained nectin-1. An increased concentration of nectin-1 in the serum was associated with poorer survival [28].

## 4. Nectin-2

There are several studies, mainly concerning gastrointestinal cancers, which suggest nectin-2 involvement in cancers’ development and the impact on prognosis (Table 3).

When studying HCC cell lines, Toutirais et al. found that nectin-2 was expressed in all of the evaluated cell lines [29]. The first study exploring the role of nectin-2 as a prognostic factor in HCC was conducted by Huang et al. This research was carried out on 159 HCC tissue samples. IHC staining revealed that 68 cancers (42.7%) were classified as high-expressing, whereas 91 tumors (57.2%) had low nectin-2 expression. Importantly, adjacent non-cancerous tissues expressed more nectin-2 than tumor tissues. The analysis of nectin-2 expression with the clinical data showed significantly lower OS among patients with low-expressing tumors (mean 27.6 months with low expression vs. 32.6 months with high expression), although regression tests showed that nectin-2 was not an independent prognostic factor [30]. Contradictory results for nectin-2 expression were reported by Ho et al. in their study concerning HBV-related HCC. The expression of nectin-2 was significantly higher in the tumor specimens compared to the corresponding liver tissue [31].

**Table 3:** Nectin-2 expression, impact on prognosis and diagnostic possibilities.

Authors	Cancer	Material	Results and Conclusions
Huang et al. [30]	HCC	159 tissue samples (cancerous and adjacent tissue)	Low expression of nectin-2 was associated with poorer OS but was not an independent prognostic factor.
Martin et al. [21]	BrC	133 breast cancer tissues, 27 control cancer-free breast tissues	Nectin-2 was reduced in tumors with positive lymph nodes and metastasis, increasing NPI and tumor grade. The expression was elevated with higher TNM (I vs. III), ER tumors and in ductal carcinoma. Patients who died from the disease had reduced expression of nectin-2. High-expressing patients had longer mean survival.
Zhang et al. [32]	CRC	42 cancer samples	Nectin-2 expressed in 52.4% samples, no association between expression and clinicopathological data.
Karabulut et al. [34]	CRC	140 serum samples	Nectin-2 concentrations were generally higher among CRC patients. Among non-metastatic patients, higher levels of nectin-2 indicated worse PFS.
Wang et al. [35]	GC	670 tissue samples	Nectin-2 was expressed in 90% of GC samples and was related to OS. The scoring system created (including nectin-2) was an independent marker of worse prognosis.
Xu et al. [36]	GC	150 tissues and blood samples, GC cell lines and TCGA data	Nectin-2 was overexpressed in GC and correlated with poorer survival of the patients.
Li et al. [37]	ESCC	106 tissue samples, cell lines	Nectin-2 was overexpressed in tumor tissue compared to normal mucosa. Over 70% of tumors expressed nectin-2 highly and moderately. Higher expression was related to features indicating poorer survival—tumor size, stage and differentiation.
Liang et al. [38]	PDAC	106 cancer tissues, 35 peritumoral tissues, 55 benign pancreatic lesions, 13 normal pancreatic tissues	Nectin-2 was expressed in 54.7% of cancers, while higher percentages were observed in more aggressive tumors. Nectin-2 is an independent prognostic factor of shorter OS.
Izumi et al. [23]	PAC	49 cancer samples	Nectin-2 was weakly expressed and correlated only with tumors’ grade. No connection with OS was established.
Bekes et al. [39]	OC	60 ovarian cancer samples, 20 healthy controls	Nectin-2 was highly expressed in OC, with especially high expression in TNM N+ tumors (indicating possible prognostic value).
Ni et al. [40]	OC	132 urine samples—73 malignant and 59 benign lesions	Classifier created (based on WFDC2, PTMA, nectin-4, FIBA, and nectin-2) to distinguish benign from malignant lesions with AUC=0.952 in ROC analysis.
Wang et al. [41]	BC	1603 patients—Surveillance, Epidemiology and End Results (SEER) database	*NECTIN2* was the only gene which could predict metastasis.
Erturk et al. [42]	LC	74 LC patients’ serum samples, 40 controls	Nectin-2 served as a biomarker with 91.9% sensitivity and 92.5% specificity in the study.

HCC—hepatocellular carcinoma; BrC—breast cancer; CRC—colorectal cancer; GC—gastric cancer; ESCC—esophageal squamous cell carcinoma; PDAC—pancreatic ductal adenocarcinoma; PAC—pancreatic adenocarcinoma; OC—ovarian cancer; BC—bladder cancer; LC—lung cancer.

In the previously mentioned study on breast cancer by Martin et al., nectin-2 expression was reduced in tumors with positive lymph nodes, increasing NPI and tumor grade. On the other hand, the expression was elevated with higher TNM (I vs. III), but again reduced in metastatic disease and in patients who had died of the disease. Increased levels of nectin-2 were detected in ER tumors and in ductal carcinoma [21].

The first broad study of nectin-2 expression in colorectal cancer was conducted by Zhang et al. Among 42 CRC samples, nectin-2 was expressed in 52.4% of tumors. No association between nectin-2 and clinicopathological data (lymph node invasion, differentiation and Duke’s stage) was found [32]. Turin et al. cultured CRC cells derived from surgically excised specimens of cancer liver metastasis (25 patients). Their results showed high expression of nectin-2 in all cancer samples [33]. Karabulut et al. studied 140 serum samples of CRC patients and measured nectin-2 levels. Compared to the control, patients with CRC had significantly higher concentrations of nectin-2 in the serum. It was determined that among the non-metastatic group of patients, those with elevated nectin-2 levels had worse progression-free survival (PFS). However, no association between serum concentration of nectin-2 and OS was found [34].

In the field of GC, there are recent studies implying that nectin-2 is involved in prognosis. Wang et al. attempted to create an immunosuppression scoring system based on immune checkpoints analysis. Only 6 out of 20 were related to OS (nectin-2, CEACAM1, SIGLEC6, adenosine, CD44 and CD155). Nectin-2 was found to be expressed in about 90% of the tissues studied. The overall result was that the scoring system created is an independent prognostic factor for GC, implying worse OS, and could be implemented as a predictive factor [35]. Xu et al. collected 150 GC tissue and blood samples from patients and enriched their study with cell lines and TCGA data set for GC. The results showed that nectin-2 was notably overexpressed in GC and was a negative prognostic factor for OS [36]. Another digestive tract cancer was evaluated by Li et al. A total of 106 esophageal squamous cell carcinoma (ESCC) patients were enrolled in the study. The expression of nectin-2 was significantly higher in the cancer tissue than the healthy mucosa. High expression was observed in 35.8% of the tumors and 39.6% expressed nectin-2 moderately. Further analysis with clinical data and the cell line study revealed that elevated expression of nectin-2 indicated a more aggressive tumor, since it correlated with tumor size, advanced tumor stage and poor differentiation [37].

Nectin-2 was also the subject of a study on PDAC. Liang et al. studied 106 cancer tissues and compared them to peritumoral tissues, benign lesions and normal pancreatic tissue. Nectin-2 was found to be expressed in 54.7% of cancers, which was a significantly higher percentage than in the control groups. Poorly differentiated cancers, tumors infiltrating surrounding tissues, cancers with metastasis to lymph nodes and TNM III and IV were identified as having higher percentages of nectin-2 expression. Finally, nectin-2 expression was correlated with shorter OS and was determined to be an independent prognostic factor [38]. In Izumi’s study on PAC, nectin-2 expression was weak and did not correlate with patients’ OS. Nectin-2 was only related to the tumors’ grade [23].

Bekes et al. found high expression of nectin-2 in ovarian cancer (OC) tissues. Particularly elevated expression was noted in patients with lymph node metastasis, therefore the authors associate it with a possible prognostic value [39]. Ni et al. developed a proteomics-derived classifier used to distinguish malignant from benign lesions of the ovaries based on urinary analysis of selected proteins involving WFDC2, PTMA, nectin-4, FIBA, and nectin-2. The area under curve (AUC) values for the classifier were 0.970 in the test dataset and 0.952 in the validation dataset, proving its promising role as a biomarker [40].

In bladder cancer, Wang et al. explored a genetic signature predicting cancer metastasis. A signature incorporating 18 genes proved highly predictive, although the *NECTIN2* gene was the only one that could predict metastasis. Thus, the authors speculated whether nectin-2 is a trigger for metastasis in bladder cancer [41].

The concentrations of nectin-2 and nectin-4 were evaluated in lung cancer (LC) patients’ blood samples by Erturk et al. The concentration of nectin-2 was higher in the group studied compared to the controls. Although only non-small cell lung cancer (NSCLC) stage was associated with nectin-2 serum levels, nectin-2 showed 91.9% sensitivity and 92.5% specificity as a LC biomarker. No prognostic value was determined [42].

## 5. Nectin-3

Nectin-3 is the least explored of all nectins. There are few reports about its prognostic value in cancers (Table 4), and no articles on the topic of HCC that would match this review criteria were found in the database.

In breast cancer, nectin-3 expression was reduced in cancers with positive lymph nodes, increasing NPI and grade, but was elevated with increasing TNM (I vs. III). However, in the presence of metastasis, the expression was reduced. ER+ tumors had a higher expression of nectin-3, while lower levels were observed among ductal carcinomas. Patients with high-expressing carcinomas had better outcomes [21].

Izumi et al. evaluated nectin-3 in PAC and although the expression was not correlated with clinicopathological characteristics of the tumors, it was linked with patients’ outcomes. The researchers based the IHC staining on scores (0 to 3 with an increasing level of nectin expression) and established that patients with score 3 tumors had overall better survival than patients with lower scores. In multivariate analysis, nectin-3 score and tumor size were independent prognostic factors [23].

Pancreatic neuroendocrine tumors (PanNET) were studied by Hirabayashi et al., whose findings suggested that a loss of nectin-3 expression was an indicator of tumor aggressiveness—larger size, G2/3, higher Ki67, lymph node metastasis, more advanced disease stage. Low nectin-3 expression correlated with several factors of worse prognosis. Patients with a loss of nectin-3 expression in the cells’ membrane had shorter disease-free survival (DFS) [43].

Nectin-3 in ovarian cancer was examined by Xu et al. Tissues from OC and non-malignant lesions were obtained and evaluated. Nectin-3 expression was detected in 26/74 OC specimens (35%), in 3/24 (12%) borderline ovarian tumors (BOT) and in 2/34 benign ovarian tumors (BOET). The positive expression rate was significantly higher in OC, while Western blot showed higher levels in OC and BOT compared to BOET. The survival data demonstrated that positive staining for nectin-3 was a prognostic factor of worse 5-year survival. Furthermore, it correlated with the tumor stage [44].

The prognostic value of nectin-3 was evaluated in lung adenocarcinoma (LAC) by Maniwa et al. In normal peritumoral lung tissue, nectin-3 was not expressed, but it was detected in 81% of cancers. In 25% of tumors, nectin-3 was expressed in the cells’ membrane. The membranous pattern of expression was demonstrated to be an independent prognostic factor of worse OS.

## 6. Nectin-4

Evidence of the prognostic value of nectin-4 is the most abundant among all nectins. Generally, overexpression of nectin-4 in cancer tissues is linked to a worse prognosis (Table 5).

Only one study concerning HCC and nectin-4 was found in the database. The mRNA and protein levels of nectin-4 were markedly higher in the tumor than in adjacent tissues. Overall, 70% (14/20) tumors proved positive for nectin-4 mRNA and 65% (13/20) tumors tested positive in Western blot. In IHC, there were 67.82% positively stained tumors. High expression of nectin-4 correlated significantly with tumor size, the presence of metastasis vascular invasion, and TNM stage. Patients with tumors expressing nectin-4 had shorter recurrence-free survival (RFS) and OS than patients with nectin-4 negative tumors. The Cox regression model revealed that nectin-4 was (along with metastatic status, vascular invasion, and TNM stage) an independent prognostic factor for shorter RFS and OS in HCC [46].

There is a wide range of studies concerning the role of nectin-4 in breast cancer. Lattanzio et al. shed light on nectin-4 expression in early BrC. A total of 197 completely resected T1-T2 tumors were evaluated in the study. Nectin-4 was expressed in the cells membranes of 34 (17%) of tumors, while cytoplasmic expression was detected in 151 specimens. It was shown that membranous nectin-4 expression correlated with shorter distant-relapse-free survival (DRFS) in the whole group and DFS in patients with luminal-A tumors, and was an independent prognostic factor. Low cytoplasmic expression was an independent prognostic factor for local-relapse-free survival (LRFS), particularly DFS in luminal-A tumors [47]. In contrast to the aforementioned research, Rajc et al. focused on luminal-B HER2-negative BrC. A total of 147 samples were studied. Of these, 78 tumors were low expressing, while high expression was detected in 69 samples and significantly correlated with tumor size, but most importantly, it was associated with worse DFS, DRFS, OS and LRFS. In the lymph nodes, a negative subgroup nectin-4 correlated with all survival models apart from LRFS. On the other hand, in lymph node-positive patients, DRFS was not correlated with nectin-4 expression. In multivariate analysis, nectin-4 proved to be a prognostic factor for OS and DFS [48].

Two other papers explored triple-negative breast cancer (TNBC) and the role of nectin-4 in assessing prognosis. Interestingly, the reported results are somewhat contradictory. In M-Rabet et al.’s study, nectin-4 was highly expressed in 62% of cases and was not present in normal tissues. High expression correlated with shorter metastasis-free survival (MFS) in the whole group, but according to the univariate analysis, it was significant only for the TN subtype and was considered a prognostic factor of poor survival [49]. Zeindler et al. reported conflicting results. Although nectin-4 was also highly expressed in a similar percentage of tumors (58%), the authors demonstrated that high expression is a predictor of better OS in patients with TNBC. It was also associated with smaller tumor size and the absence of lymph node metastasis [50].

In their consecutive studies, Sethy et al. focused on invasive ductal carcinoma (IDC) and the role of nectin-4 in metastatic progression. In the first study, nectin-4 was strongly expressed in 92% of samples (but was present in all specimens). It correlated with lymph node metastasis, tumor size and grade [51]. In the other study, the authors report an interesting finding of a higher expression of nectin-4 among patients using tobacco and/or alcohol. Correlations between expression and patients’ occupation were also reported [52].

Urothelial carcinoma is one of the best-studied cancers in terms of nectin-4, given the available treatments based on targeting nectin-4 in this malignancy [53]. In the latest studies, Tomiyama et al. report expression of nectin-4 in 65.7% of the upper tract urothelial carcinomas (UTUC) studied. Strong nectin-4 expression was shown to be a risk factor for disease progression and increased disease-related mortality [54].

**Table 5:** Nectin-4 expression and impact on prognosis.

Authors	Cancer	Material	Results and Conclusions
Ma et al. [46]	HCC	20 tumor specimens and adjacent non-tumor fragments (mRNA and protein—Western blot) 87 tumor samples (IHC)	Nectin-4 was expressed in ~65–70% of tumors and positive nectin-4 status was an independent prognostic factor for shorter RFS and OS.
Lattanzio et al. [47]	BrC	197 tumor samples	A membranous pattern of nectin-4 expression was an independent prognostic factor of worse DFS in luminal-A BrC. Low cytoplasmic expression was also a prognostic factor influencing LRFS in general and DFS in luminal-A tumors.
Rajc et al. [48]	BrC	147 tumor samples	High nectin-4 expression was associated with all survival models, but was established as a risk factor for OS and DFS in luminal-B tumors.
M-Rabet et al. [49]	BrC	353 samples (for DNA microarrays) 61 samples (for IHC) 5673 patients’ data (from an outside database)	High nectin-4 expression was a negative prognostic factor for MFS in patients with TNBC.
Zeindler et al. [50]	BrC	148 tumor samples	High expression of nectin-4 predicted better survival, and was associated with favorable pathological features in TNBC.
Sethy et al. [51]	BrC	100 tumor samples	Nectin-4 was highly expressed in 92% of samples and was linked to unfavorable disease characteristics—lymph node metastasis, tumor size and grade.
Tomiyama et al. [54]	UTUC	99 tumor samples	Nectin-4 expressed in 65.7% of samples and was a negative prognostic factor of PFS, and was linked to higher mortality.
Zhang et al. [55]	CRC	372 CRC samples and 31 normal samples from TCGA 68 CRC and 15 normal paraffin samples	Nectin-4 showed high expression in most of the tumors and suggested potential prognostic value, because it correlated with TNM stage, lymph nodes and distant metastasis (but not T stage).
Deng et al. [56]	EC	TCGA datasets 94 cancer tissues and 78 normal tissues	Nectin-4 had strong expression in EC in IHC staining, but a low mRNA expression. Both aforementioned features indicated worse OS, but a higher expression in IHC was significantly correlated with OS and was suggested as a prognostic factor.
Lin et al. [57]	EC	94 cancer specimens	Nectin-4 was mostly overexpressed in cancer, and higher expression correlated with worse OS and was a prognostic factor.
Zhang et al. [58]	GC	20 paired cancer/healthy tissues 303 cancer specimens 91 adjacent normal tissues	Nectin-4 was expressed in 60% of cancers. High expression was a negative prognostic factor for OS.
Zhang et al. [59]	GC	64 paired cancer/healthy samples	Nectin-4 was highly expressed in 70% of samples and was correlated with shorter 5-year survival.
Nabih et al. [60]	OC	39 patients with OC 21 patients with benign lesions (25 control benign biopsies)	Nectin-4 was increased in malignant compared to benign lesions. Combined nectin-4 mRNA with CA-125 showed the highest sensitivity in discriminating malignant from benign tumors. Nectin-4 was suggested as a marker for diagnosis and treatment monitoring.
Bekos et al. [61]	OC	90 samples of HGSOC	High expression of nectin-4 was an independent prognostic factor of worse OS.
Hao et al. [62]	PTC	19 + 44 thyroid cancer tissues with adjacent tissue	Nectin-4 was upregulated in tumors and correlated with disease stage (and N stage), tumor size and histological type.
Toda et al. [63]	ATC	54 samples	Nectin-4 was highly expressed in tumors and was associated with more aggressive cancers.
Tanaka et al. [64]	Melanoma	126 melanoma samples	A high level of nectin-4 was significantly associated with BRAF mutation and worse DFS, melanoma-specific survival and OS, and therefore also indicated poor prognosis.
Murata et al. [65]	EMPD	110 EMPD samples	Up-regulated nectin-4 was associated with higher TNM and thicker tumors, but had no impact on survival.
Erturk et al. [42]	LC	74 LC patients’ serum samples, 40 controls	The serum nectin-4 was elevated in LC patients and correlated with NSCLC stage, the tumor size and metastasis, but was not prognostic.
Zhang et al. [66]	GBC	68 cancer samples 60 cholecystitis tissues	Nectin-4 was an independent prognostic factor and indicated poorer OS in GBC. Overexpression correlated with T stage and lymph nodes metastasis.
Nishiwada et al. [67]	PDAC	123 cancer samples	Higher nectin-4 expression was associated with increased Ki-67 and poorer survival. Multivariate analysis showed that overexpression of nectin-4 was an independent prognostic factor.

HCC—hepatocellular carcinoma; BrC—breast cancer; UTUC—upper tract urothelial carcinoma; CRC—colorectal cancer; EC—esophageal cancer; GC—gastric cancer; OC—ovarian cancer; PTC—papillary thyroid cancer; ATC—anaplastic thyroid carcinoma; EMPD—Extramammary Paget’s disease; LC—lung cancer; GBC—gallbladder cancer; PDAC—pancreatic ductal adenocarcinoma.

On the topic of CRC, Zhang et al. reported upregulated expression of nectin-4 in tumor tissues compared to normal mucosa. High expression correlated with lymph node and distant metastasis and was also associated with disease stage (TNM), but not with T feature. Additionally, 70.6% of the samples revealed high expression in IHC staining. It was concluded that due to the association of nectin-4 expression with the clinicopathological markers of worse prognosis, nectin-4 could serve as a potential prognostic marker, especially for identifying disease progression [55].

Deng et al. reported a higher expression of nectin-4 mRNA in the normal esophageal tissue than in the esophageal cancer (EC). Furthermore, low levels of mRNA were associated with better OS (yet the difference was not significant). However, in IHC staining, nectin-4 showed strong expression in the cancer tissue, whereas there was weak or no staining in the adjacent tissues. The overexpression in cancer was significantly associated with worse OS, and it was suggested that nectin-4 could be a prognostic factor in EC [56]. Lin et al. reached similar conclusions in their study, in which nectin-4 was also highly expressed in cancer tissue at the protein level. However, in contrast to the previously mentioned research, nectin-4 was also overexpressed at the mRNA level. Higher expression served as a prognostic factor of poor OS [57].

The prognostic role of nectin-4 in GC was first evaluated by Zhang et al. Its expression was detected in 60% of cancer tissues and 15% of normal gastric tissues. Patients with high expression had poorer OS rates and nectin-4 proved to be an independent prognostic factor [58]. In another study on GC, nectin-4 was also found to be upregulated in cancer compared to the adjacent tissues and was highly expressed in 70% of samples. Patients with high nectin-4 expression had markedly shorter 5-year survival [59].

Nabih et al. studied different histological subtypes of OC. Nectin-4 was evaluated in tissues and serum, and in both materials, it was significantly increased in malignant compared to benign lesions, especially in more advanced tumors. The authors attempted to create models with cut-off values of markers to distinguish benign from malignant lesions. The sensitivity of nectin-4 mRNA was highest among the three markers and reached 97.4%. The combined CA-125 and serum nectin-4 had lower sensitivity than CA-125 and nectin-4 mRNA expression. CA-125 and nectin levels were significantly correlated. Similarly, there was a significant association between serum nectin-4 and nectin-4 mRNA expression. The authors concluded that nectin-4 could serve as a marker for distinguishing OC from benign lesions and for monitoring the cancer treatment [60]. In high-grade serous ovarian cancer (HGSOC), nectin-4 was also found to be upregulated. High expression was associated with more neoadjuvant chemotherapy being introduced, but was not correlated with platinum-based chemotherapy resistance. Nectin-4 was proved to be an independent negative prognostic factor of OS [61].

In patients suffering from papillary thyroid cancer (PTC), nectin-4 was found to be markedly expressed in tumors, but less so in the adjacent tissues. A high expression correlated with disease stage (and lymph node metastasis), tumor size and histological type. Therefore, it is plausible to extrapolate that it might have potential prognostic value [62]. Toda et al. evaluated anaplastic thyroid carcinoma (ATC). The authors found that 59% of ATC was expressing nectin-4. The tumors with overexpression of nectin-4 had significantly quicker growth, which implies more aggressive tumors. Moreover, nectin-4 was higher in de novo types of ATC [63].

Nectin-4 showed high positive staining in 35.7% of melanoma samples. High expression was associated with BRAF mutation and significantly correlated with poorer DFS, melanoma-specific survival and OS [64]. Murata et al. found positive staining for nectin-4 in normal skin tissues, while in extramammary Paget’s disease (EMPD) 37.3% were highly expressing lesions. More advanced TNM and greater tumor thickness correlated with high expression of nectin-4. However, no impact of disease-specific survival was exposed [65].

As mentioned earlier, serum nectin-4 was studied in LC. The results show elevated nectin-4 in LC patients compared to controls. The serum level correlated with NSCLC stage, tumor size and the presence of metastasis, but it was not found to be prognostic. Its diagnostic accuracy was also notably lower than that of nectin-2 [42].

Zhang et al. compared nectin-4 expression in gallbladder cancer (GBC) and cholecystitis-affected gallbladders. In 63.2% of GBC, nectin-4 was upregulated, but only 1.7% of cholecystitis samples showed positive nectin-4 staining. Expression of nectin-4 correlated with T stage and lymph node metastasis in GBC. Nectin-4 was an independent prognostic factor for GBC and was associated with significantly shorter OS [66].

Nectin-4 showed abundant expression in PDAC: 56.1% of tumors were classified as high-expressing cancers. Patients with overexpressed nectin-4 had poorer survival rates and a higher percentage of recurrences than low-expressing patients. Tumors with upregulated nectin-4 also had higher levels of Ki-67. Nectin-4 in multivariate analysis was shown to be an independent prognostic factor for PDAC [67].

## 7. Discussion

In the articles presented above, there is significant evidence which proves that nectins have considerable potential to become novel markers for prognostic calculations in multiple cancers. There are little data available regarding the diagnostic utility of nectins; therefore, this topic will require more studies. However, there are projects in progress and already published research evaluating the use of nectins in treatment.

An interesting direction for cancer treatment where nectins are involved lies in virotherapy. There are several reports of virus-based therapy mostly focusing on measles virus and its entry receptor, nectin-4. Researchers exploited high nectin-4 expression rates in breast cancer and attempted to develop an oncolytic measles virus selectively blind to the signaling lymphocyte activation molecule (SLAMblind). The studies showed promising oncolytic activity in vitro and in vivo in xenografts by decreasing the viability of cancer cells and by suppressing the growth of tumors. Moreover, the virus was considered safe and free from measle-like symptoms [68,69]. Further studies also explored SLAMblind measles virus activity in pancreatic cancer, LC and CRC, and reported similar results of the virus oncolytic activity in vitro and its ability to suppress tumor growth in xenografts [70,71,72]. Nectin-1 is a viral entry receptor for herpes simplex virus (HSV). Like measles virus, HSV was engineered into a therapy agent. Studies on different neoplasms—like squamous cell carcinoma, gliomas, melanoma, myeloma and some hematological malignancies—suggest that oncolytic HSV has the potential to cause disease regression and might be considered as a treatment option in the future [73,74,75,76,77].

Although the perspective of virotherapy is compelling, the main efforts are focused on clinical trials with antibody–drug conjugate (ADC) therapeutics, the most famous of which is Enfortumab vedotin (EV). It was designed as a human anti-nectin-4 antibody conjugated with monomethyl auristatin E (MMAE), which is a microtubule-disrupting agent. The EV proved its efficacy in preclinical animal xenograft models in urothelial cancer and breast cancer [53]. Several reports from clinical trials seem to confirm that EV is a quite well-tolerated drug with a documented response to treatment—also in locally advanced and metastatic urothelial cancers. EV is considered a new next-line treatment for patients with urothelial cancer who have received platinum and anti-PD-1/L1 treatments and are left with few therapeutic options. However, some on-target toxicity effects, especially those affecting skin, are observed during therapy, such as rash, alopecia, pruritus and dry skin [78,79,80,81]. In 2021, the Food and Drug Administration approved EV as a treatment for patients with urothelial cancer who were treated with platinum and anti-PD-1/L1, and were ineligible for cisplatin therapy. M-Rabet et al. also developed ADC targeting nectin-4, which showed considerable activity against TNBC animal xenografts in primary tumors, metastasis and local relapse [49].

Shao et al. approached and exploited the expression of nectin-4 in breast cancer differently to previously mentioned researchers. The authors synthesized a radionuclide probe ^99m^Tc-HYNIC-mAb_Nectin-4_ to assess nectin-4 expression in breast cancer xenografts in mice via immunoSPECT/CT imaging. Furthermore, a photothermal agent mAb_Nectin-4_-ICG was developed to conduct targeted photothermal therapy of the tumors, which resulted in significant suppression of the tumors [82].

A step ahead from ADC seems to have been achieved with the development of the Bicycle^®^ technology using bicyclic peptides capable of conjugating with other compounds. One of them is BT7480—Bicycle tumor-targeted immune cell agonist™ targeting nectin-4 and CD137. The condition for the drug’s activity is binding to nectin-4 and CD137 at the same time, so the effect is elicited only in nectin-4-positive tumors. The agonism to CD137 causes activation of immune cells, disrupting the tumor’s microenvironment and reprogramming to upregulation of cytotoxicity-related genes. In the study cited, BT7480 showed complete regression of nectin-4-positive tumor xenografts models with resistance to tumor relapse [83]. In another study, BT8009 Bicycle^®^ toxin conjugate targeting nectin-4 with the same cytotoxic substance as in EV showed similar or superior results compared to EV in preclinical models. According to the authors, clinical trials with BT8009 are ongoing [84,85].

The close relations of nectins to TIGIT, CD96 and CD226 make them an interesting target for immunotherapy of cancers. Blocking TIGIT showed promising anti-tumor effects in pre-clinical studies. Since treatments targeting PD-1/PD-L1 are already available and have proved effective, it is reasonable to continue developing new immunotherapies [14].

## 8. Conclusions

Systematic reviews have some limitations, which include the risk of bias. The selection of studies and decision on the relevance of the content is based on human judgment. We strived for accuracy and honesty in selecting the studies that were included.

In the articles analyzed, there are few data regarding the usefulness of nectins in specifying prognosis or improving the diagnosis of HCC. In fact, we have found single reports concerning nectin-1, nectin-2 and nectin-4, which show the scope for related research and emphasize the need for further studies on this topic. Although each of the articles quoted give the premises for the possible utility of nectins in HCC, the number of studies precludes strong conclusions. HCC is a neoplasm with a bleak prognosis for a number of patients, therefore developing new diagnostic methods and finding new prognostic factors seems to be of high importance. Our observation from the studies that were reviewed is that most reports did not compare nectin expression with other typically used markers. Such a comparison needs to be assessed in order to determine whether using nectins as differentiating elements, prognostic factors and diagnostic markers is superior to the current methods. At present, the most extensive evidence is available for urothelial and breast cancer, but the amount of data on ovarian cancer and pancreatic cancer is increasing. Since treatments based on nectin-4 expression in some tumors are being launched, there is considerable potential to develop new therapies for neoplasms like HCC or CRC in the near future.

Nectin-1 was mostly correlated with a worse prognosis when upregulation was present. The opposite results were seen in a minority of studies. Similarly, nectin-2 was generally overexpressed in tumors, which was associated with poorer outcomes. In a couple of studies, low nectin-2 expression marked worse prognosis. Nectin-3 is the least-studied member of the family, with contradictory results. A loss of expression or overexpression could indicate worse outcomes, depending on the cancer. The expression patterns of nectin-4 were most consistent. Generally, nectin-4 tends to be upregulated in multiple tumors and has the best documented prognostic value. On that account, it is most promising as a potential marker and a target for treatment.

## Figures and Tables

**Table 1:** Nectin family characteristics [9].

Nectin	Binding Partners	Function
Nectin-1	nectin-1, nectin-3, nectin-4, necl-1, CD96	Cell adhesion, immune modulation, viral entry receptor
Nectin-2	nectin-2, TIGIT, CD226	Cell adhesion, immune modulation, viral entry receptor
Nectin-3	nectin-3, nectin-1, nectin-2, necl-2, necl-5, TIGIT	Cell adhesion, immune modulation
Nectin-4	nectin-4, nectin-1	Cell adhesion, viral entry receptor

**Table 4:** Nectin-3 expression and impact on prognosis.

Authors	Cancer	Material	Results and Conclusions
Martin et al. [21]	BrC	133 breast cancer tissues, 27 control cancer-free breast tissues	Expression was reduced in more advanced tumors. Patients with higher expressing tumors had better outcomes.
Izumi et al. [23]	PAC	49 cancer samples	Higher expression of nectin-3 was an independent prognostic factor of better OS.
Hirabayashi et al. [43]	PanNET	78 tumor tissue samples	Loss of nectin-3 expression was a prognostic factor of worse prognosis—shorter DFS—but it was not statistically significant in multivariate analysis.
Xu et al. [44]	OC	74 OC tissue specimens 24 BOT tissues 34 BOET tissues	Nectin-3 was expressed in 35% of OC specimens and was a risk factor of shorter survival.
Maniwa et al. [45]	LAC	127 cancer tissues	Nectin-3 was expressed in 81% of cancers and in 25%, it was expressed in the membranes, which was an independent negative prognostic factor.

BrC—breast cancer; PAC—pancreatic adenocarcinoma; PanNET—pancreatic neuroendocrine tumor; OC—ovarian cancer; LAC—lung adenocarcinoma.
